# Production activities and economic dependency by age and gender in Europe: A cross-country comparison

**DOI:** 10.1016/j.jeoa.2014.09.007

**Published:** 2015-04

**Authors:** Bernhard Hammer, Alexia Prskawetz, Inga Freund

**Affiliations:** a Vienna University of Technology, Institute of Mathematical Methods in Economics, Argentinierstrasse 8/4/105-3, 1040 Vienna, Austria; b Wittgenstein Centre for Demography and Global Human Capital (IIASA, VID/ÖAW, WU), Vienna Institute of Demography/Austrian Academy of Sciences, Austria

**Keywords:** National, Economic dependency ratio

## Abstract

We compare selected European countries using an economic dependency ratio which emphasizes the role of age-specific levels of production and consumption. Our analysis reveals large differences in the age- and gender-specific level and type of production activities across selected European countries and identifies possible strategies to adjust age-specific economic behaviour to an ageing population. The cross-country differences in economic dependency of children and elderly persons are largely determined by the age at which people enter, respectively exit, the labour market. The ability of the working age population to support children and elderly persons in turn is strongly influenced by the participation of women in paid work. We also provide a measure for the age-specific production and consumption in form of unpaid household work. The inclusion of unpaid household work leads to a decrease of the gender differences in production activities and indicates that the working age population supports children and elderly persons not only through monetary transfers but also through services produced by unpaid work (e.g. childcare, cooking, cleaning…). Given the available data, we cannot distinguish the age profile of consumption by gender and have to assume – in case of unpaid work - that each member of the household consumes the same. Hence, our results have to be regarded as a first approximation only. Our paper aims to argue that a reform of the welfare system needs to take into account not only public transfers but also private transfers, in particular the transfers in form of goods and services produced through unpaid household work.

## Introduction

Persistent low fertility and increasing survival to older ages are the key determinants of population ageing in many European countries. The consequences of the changing age structure for the overall economic development depend on the design of the economic life cycle, i.e. the age pattern of economic activities such as consumption, the generation of labour income and saving. A typical characteristic of the life cycle in modern societies are phases of economic dependency at the beginning and end of life, in which consumption exceeds the income generated through one’s own labour input. In childhood and retirement at least part of consumption has to be covered through the reallocation of resources in form of transfers and asset accumulation. A shift in the age structure of the population - as a consequence of the ageing process - requires an adjustment of the age reallocation system. The current system will be under pressure as an increasing share of elderly people has to be sustained by an ageing and shrinking population in working age. The shift in the age structure of the population will be remarkable: according to EUROSTAT projections the population of the European Union aged 20–64 decreases from 308 m in 2013 to 289 m in 2030, while the population aged 65+ increases from 92 m in 2013 to 124 m in 2030.^2^

In this paper we analyse the cross-country differences in the age- and gender-specific involvement in production activities. These differences are influenced by country-specific institutional settings, practices and norms as well as the current demographic structure. With the comparative analysis we aim to identify challenges, but also possible strategies and best practice examples regarding the organisation of production and the reallocation of resources across age. We argue that a better understanding of the reallocation of resources across age is necessary to guide any welfare reform in the face of population ageing. In particular it needs to consider gender differences in the type and the intensity of production activities at each age as well as private transfers in combination with public transfers. For instance, the involvement of women in paid work might alleviate the financing of public transfers to children and dependent elderly persons. However, since women take up a great share of unpaid work, any reform that aims to increase female labour force participation also needs to consider that such a reform may reduce female’s contribution to unpaid work.

The analysis is based on the methodology and data from the National Transfer Accounts (NTA) project, as well as on income data from the European Survey of Income and Living Conditions (EU-SILC) and data from the Multinational Time Use Study (MTUS), complemented by Austrian time use data. From these data sources we obtain information on the age-specific levels of production^3^ and consumption. The difference between consumption and labour income is termed the *life cycle deficit* (LCD) (Mason et al., 2006) and represents a measure for the age specific level of economic dependency. For children as well as for elderly persons the life cycle deficit is positive, i.e. average consumption in these ages exceeds average labour income. The LCD is negative during the working years when labour income is higher than consumption. For a negative life cycle deficit we will also use the term *life cycle surplus* (LCS). By multiplying the age-specific per capita LCD with the corresponding population numbers and summing up over all age groups with a positive LCD, we obtain a measure for the total economic dependency of children, respectively elderly persons. The total economic surplus of the working age population (the sum over the age groups with a negative LCD) gives us a measure for a society’s ability to support the population with a (positive) life cycle deficit. Different to the commonly used demographic measures, like the standard demographic young and old age dependency ratios,^4^ that are based on fixed age limits and consider only the demographic structure, the aggregate life cycle deficit allows for flexible age limits and age-specific levels of economic dependency. NTA data therefore allow to endogenously define the stages of the life cycle. The importance of such measures is emphasized in Sanderson and Scherbov (2013), who argue that that a focus on chronological age limits the insight into the process of population ageing.

In Section “The life cycle deficit for paid work” we give an overview of the NTA methodology and present the LCD as a measure of economic dependency for selected European countries. In Section “The life cycle deficit by gender” the LCD and LCS are presented for men and women separately. Since our emphasis is on the role of the age specific design of the economic life cycle independent of the demographic structure, we control for cross-country differences in the population structure by applying a standardized population for all countries. With this analysis we gain further insights into the cross-country differences regarding the gender-specific shape of the economic life cycle. By only considering paid work the estimates for production activities by gender are biased since they ignore unpaid household labour that is on average higher for females as compared to males. We therefore further extend our analysis by unpaid household work in Section “Unpaid work” and build up an indicator that measures the difference between the production and consumption of goods and services which are produced by unpaid household work in a specific age group. In Section “The life cycle deficit for paid- and unpaid work” we combine paid work as well as unpaid household work into a measure for total production and consumption at each age and by gender. Section “Conclusions” concludes.

## The life cycle deficit for paid work

### National transfer accounts

The concept of the life cycle deficit and the data on age-specific consumption are taken from the National Transfer Accounts (NTA) project which extends the System of National Accounts (SNA) by information on age - the so-called National Transfer Accounts. NTA measure how much labour- and asset income each age group generates, how income is subsequently redistributed across age groups through public and private transfers and how each age group uses the disposable resources for consumption and saving. The NTA data set consists of an extensive number of age profiles containing per capita averages of labour income, asset income, public transfers, private transfers, consumption and saving. The broad estimation strategy for age-specific averages of economic quantities is, first, to derive the aggregate values (e.g. total income, total consumption) from the System of National Accounts and related sources. In the second step the distribution of these quantities over age groups is measured or estimated by using administrative and survey data. A detailed introduction to the methodology is given in UN (2013) and in Lee and Mason (2011). The NTA project is a collaborative work of international research teams from 41 countries.^5^ Among these countries are the following 12 European countries: Austria, Finland, France, Germany, Hungary, Italy, Poland, Slovenia, Spain, Sweden, Turkey and the UK. Due to data availability we focus on 10 European countries excluding Poland and Turkey.^6^ NTA measure the economic activities of individuals in a given year. It is important to note that the age patterns represent a cross-sectional snapshot of the economic activities of each age group and do not represent the actual life course pattern of an average individual.

### The life cycle deficit

NTA are based on an accounting identity which states that for each individual, and for each age group, the resources used for consumption (*C*) and saving (*S*) equal the disposable income composed of labour income (YL), asset income (YA) and net transfer inflows (τ)^7^:

(1) $$C+S=\underset{\text{disposable}\;\text{income}}{\underset{︸}{\text{YL}+\text{YA}+\tau }}$$

The difference between consumption and labour income in NTA offers a measure for the average economic dependency (if positive) or the economic ability to support others (if negative) at each age. It can also be derived by an rearrangement of the terms in the NTA accounting identity (1):

(2) $$\underset{\text{life}\;\text{cycle}\;\text{deficit}}{\underset{︸}{C-\text{YL}}}=\underset{\text{age}\;\text{reallocations}}{\underset{︸}{\tau +(\text{YA}-S)}}$$

In childhood and old age labour income falls short of consumption. On the other hand, an average person in working age generates more income than needed for his/her own consumption. The life cycle deficit is therefore positive in childhood as well as for elderly persons and negative for the population in working age. This qualitative pattern of the economic life cycle is similar in all countries (see also Lee and Mason, 2011): the economic needs of children and elderly persons are financed through asset based reallocations and through the transfer of the surplus income from the working age population. However, the type and intensity of economic activities at each age, and therefore also the shape of the age profiles, differ across countries depending on country-specific characteristics of individuals (such as the level and type of education, labour market entry and exit ages, etc.), institutional arrangements (family policies, labour market regulations, etc.) as well as the overall macroeconomic situation of a country.

As indicated in the previous section, in order to obtain a measure for the dependency of the total population in childhood and old age, the life-cycle deficit at each age is multiplied with the corresponding population size and added up over those age-groups with a positive LCD. A dependency ratio is then calculated by relating the total life cycle deficit of the children and the elderly to total labour income. The *aggregate life cycle deficit* measures the consumption of children and the elderly which cannot be financed out of their own labour income as a share of total labour income. This measure reflects both, the population structure as well as the design of the economic life course, i.e. the involvement in production and consumption activities.^8^ Likewise we can derive a support ratio by relating the total life cycle surplus (the negative life cycle deficit) of those in working age to total labour income in order to receive the *aggregate life cycle surplus*. It represents the share of labour income which is not consumed by the working age population and available for transfers to other age groups.

### Data

The aggregate quantities are derived from the SNA. The basic components of labour income are the compensation of employees (incl. gross wages as well as the employers’ social contributions) and self-employment labour income, i.e. the part of mixed income which is assumed to be generated by labour input.^9^ Consumption consists of public consumption as well as private consumption at basic prices (i.e. without taxes on products such as the VAT). The information on the distribution of labour income by age and sex is taken from the European Survey of Income and Living Conditions (EU-SILC) 2011.^10^ This survey is carried out yearly and includes representative and comparable income data for private households for all EU member countries. The components of income which are of interest for us are the gross remuneration of employees, the employers’ social contributions and gross income from self-employment. These income components are reported as the annual income generated during the income reference period.^11^

A limitation of our data is the fact, that we do not have data on age-specific consumption by gender and that the information on consumption is not available for the same year as on labour income.^12^ The estimation of age averages for consumption is highly complex as both, public consumption as well as private consumption, consist of many different components for which often only limited age-specific information is available. Consumption age profiles have been estimated by the country teams within the NTA project.^13^ The use of consumption age profiles from different years should not affect our results: historical NTA data show that the shape of the age profiles changes only slowly with time (see e.g. Hammer, 2014 for Austria from 1995 to 2010). Furthermore, consumption of adults is rather constant over the whole adult age range. Although there is intensive work on gender-specific NTA, consumption age profiles by sex are not available for all of the countries so far. Data from those countries for which gender-specific consumption profiles are available show, that there are only small gender differences for private consumption. Some differences between men and women are found for public consumption expenditure in the categories health and long term care (see e.g. Zannella, 2013), but compared to cross-country differences the gender differences are small. We therefore assume that consumption does not differ between men and women and use the age averages provided by the NTA project for both, men and women. The consumption and labour income age profiles are adjusted so that the aggregate value of consumption and labour income corresponds to the one derived from the SNA for 2010 (Table A.1 in the Appendix).

The life cycle deficit in young and old age as well as the life cycle surplus for the European NTA countries together with the age borders when those indicators switch their sign are shown in Table 1. The table also shows the commonly used demographic dependency ratios that are based on fixed age limits and ignore the heterogeneity of economic activities over age: the demographic young age dependency ratio is calculated as the share of the population younger than 20 to those aged 20–64 years, and the old age dependency ratio as the share of the population aged 65+ to those aged 20–64 years. Obviously this indicator gives only a limited and biased estimate of the economic dependency. It neither takes into account the degree of economic dependency nor the degree of the ability to support others. The life cycle deficit in turn reflects the age structure of the population as well as age-specific labour income and consumption. A major advantage of the life cycle deficit is, that the age borders between the life cycle stages of dependency and support are not fixed but endogenously determined by the age profiles of consumption and labour income. According to this measure an average young person stays economically dependent for around 5 years longer (up to age 23–26 as indicated by the lower age borders in Table 1) than assumed in the demographic dependency ratios where the life cycle stage of young dependent people has been assumed to be delimited by age 20 (often the even lower age-border at the age of 15 is used). In old age individuals become economically dependent again about 6 years earlier (in most countries around age 59 as indicated by the upper age borders in Table 1) as compared to the assumed age limit of 65 years for the demographic dependency ratio.

Obviously, the life cycle deficit/surplus is strongly influenced by the age structure: France as the country with the highest demographic young age dependency ratio (42%) is also the country with the highest LCD in young age (29%). Italy and Germany are the countries with the highest demographic old age dependency rates (33% resp. 34%). These are also the countries with the highest LCD in old age, corresponding to 32% and 30% of total labour income, respectively. But the values for Sweden make clear that the population structure is not the only determinant of economic dependency (see also Hammer and Prskawetz, 2013): with a demographic old age dependency ratio of 31% Sweden has a rather old population. However, the LCD in old age is with 25% not particularly high. The demographic structure is compensated by a higher labour force participation and the higher labour income of elderly persons: in Sweden the average labour income exceeds the average age-specific consumption until the age of 63 years, which is 4 to 6 years longer than in the other countries. There are marked differences in the LCS across the analysed countries: while the working age population in Slovenia and Sweden uses 39% of its labour income for saving or transfers to other age groups the corresponding value is only 23% in the UK. In the following section we will investigate these differences across European countries in more depth by considering gender specific life cycle deficits and surpluses. Our aim is to focus on the design of the economic life course as it is given by the age specific characteristics of consumption and production. We therefore control for the demographic structure by applying a standardized age structure in the subsequent analyses.

## The life cycle deficit by gender

The aggregate life cycle deficit constitutes certainly an improvement for measuring economic dependency as compared to standard demographic dependency ratios that ignore the cross-country heterogeneity of economic characteristics by age. We gain further insight into the structure of economic activities at each age by calculating the life cycle deficit for men and women separately.

Since the focus of our paper is on the differences between the age specific shape of the economic life cycle across countries we apply the same standardized population age structure for all of the countries.^14^ The differences in the gender-specific life cycle deficit/surplus across countries can therefore be attributed to the differences in the shape and the level of the consumption and labour income age profiles. To obtain a compact measure to compare the age-profiles of production and consumption across countries we use the aggregate LCD/LCS.

An important determinant of the LCD/LCS is the amount of total consumption relative to total labour income. Total consumption exceeds total labour income in all of the analysed countries, as part of consumption is financed through asset income and dissaving. The ratio of consumption to labour income is influenced by the share of asset income relative to total income and by the saving rate. It is rather low in Sweden and Austria as these are countries with high saving rates (Table A.1). Thus, a large part of asset income is saved/reinvested and only a small part used for consumption. The rather low level of consumption relative to labour income in Slovenia is a result of a low share of asset income (relative to total income) and a moderately high saving rate. The high values of consumption relative to labour income for the other countries can be explained through a combination of a low/moderate saving rate of the private sector and large dissaving of the public sector (in particular in the UK, Spain, France and Hungary). Italy is an extreme case with a negative saving rate - consumption exceeds labour and asset income altogether. The result is a very high ratio of consumption to labour income and consequently a comparatively large life cycle deficit and low life cycle surplus.

**Fig. 1:**
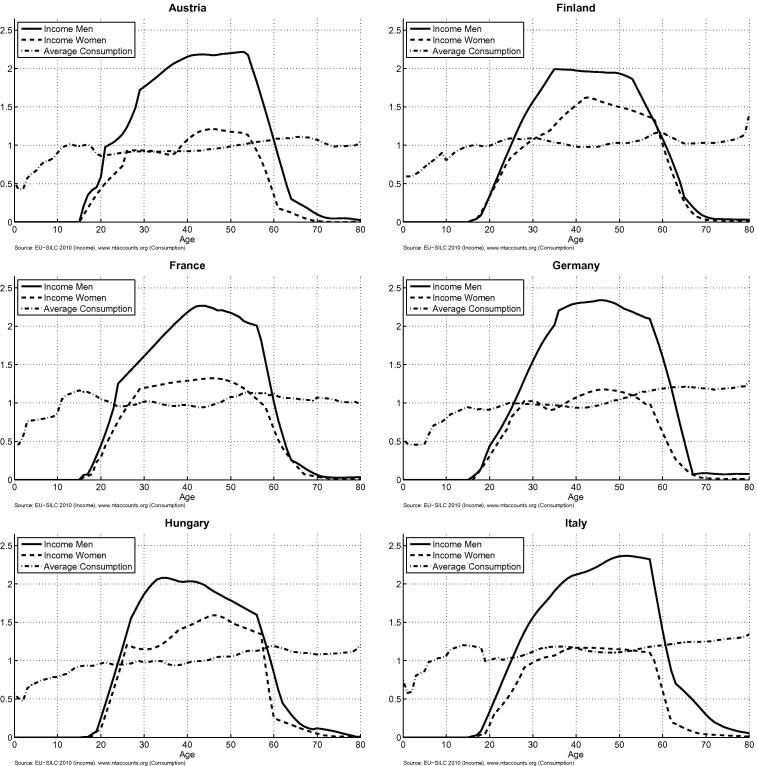

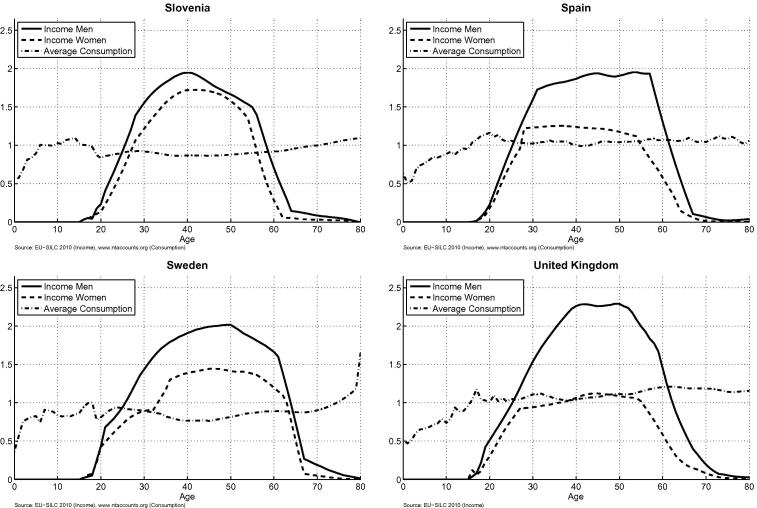
Labour income and consumption by age and sex in relation to the EU-SILC sample average of labour income.

The age-specific averages of labour income and consumption are plotted in Fig. 1. To facilitate the comparison of the age patterns across countries the age group averages are measured relative to the average income in the respective country sample, which is representative for the population aged 16+ living in private households. All profiles are smoothed to remove the random variation in the estimates of the age-specific means. The shape of the consumption age profiles is rather similar across countries, with the consumption of adults being rather constant over the age range. An exception is Sweden with a strong increase of consumption from age 70 onwards, which can be attributed to Sweden’s comprehensive but expensive system of long-term care (see Bengtsson, 2010). Two further specific consumption patterns are the fairly high average consumption of children in Italy, Slovenia and France as well as the high consumption of persons 56+ in Germany and Hungary.

The age-specific levels of labour income are clearly among the main determinants of the LCD/LCS. Particularly important are the ages at entry and exit from the labour force. In Austria young males start generating income at a younger age than in the other countries, but otherwise the income age profiles for men in young age are quite similar across countries. For young women the differences are larger, reflecting cross-country differences in female enrolment rates in higher education as well as cross-country differences in the age at which they give birth to children and their economic behaviour after giving birth. In Italy and the UK average labour income of women hardly reaches the consumption level even in the age between 40 and 50, when participation rates are high. In Hungary, Slovenia and France on the contrary average labour income of women exceeds their average consumption level already around the age of 25. For both, men and women, there are considerable cross country differences in the age group from 55 to 64 (see also the age borders in Table 1). In Slovenia, Hungary and Austria most people leave the labour market between the age of 56 and 60, reflected in the strong decline of the labour income age-profiles in these age groups. In Sweden on the other hand most of the 60 year old persons are still in the labour force, with the effect that the labour income age profiles declines at a much higher age than in other countries. Sweden is an extreme example, but also in the UK people between 60 and 70 generate a considerable amount of labour income. The most pronounced differences across countries are in the share of the labour income generated by women as compared to the labour income of men. As it is visible in Fig. 1, in all of the countries the average labour income of women is lower than that of men. But while the gender difference in Slovenia is rather low, there are large differences in Austria, Germany, Italy and the UK: the labour income of women amounts to only about one third of total income in the latter countries but is about 42 percent in Sweden and Hungary, 44 percent in Finland and 45 percent in Slovenia.^15^

With this overview of the level and the distribution of income and consumption by age and sex we can next investigate the aggregate life cycle deficit/surplus by gender shown in Table 2. Differences to the results shown in Table 1 can be ascribed to the population structure. When using the standard population Sweden is the country with the lowest LCD in old age, amounting to 21% of labour income. This indicates that the economic life cycle in Sweden has been adjusted to the comparably old population (the demographic old age dependency ratio is with 31% the third highest among the analysed countries - see Table 1). Italy on the contrary also has a high demographic old age dependency ratio (with 33% the second highest after Germany), but is also among the countries with the highest LCD in old age (29%) after controlling for the age structure effect. Furthermore, Italy is also the country with the highest LCD in young age (30%). These results reflect the high level of consumption in Italy that will be unsustainable in the long run. Austria and Germany are the countries with the lowest LCD in young age (20% of total labour income). These results are driven by the rather low average consumption of children and by the early entrance into the labour market. There are huge gender differences across countries in the generation of the life cycle surplus: the aggregate LCS ranges from 23% in the UK and 24% in Italy to 39% in Slovenia and 40% in Sweden. These differences can be attributed to the differences in the contribution of women to total labour income. While the aggregate LCS of women is virtually zero in Italy and the UK, it amounts to 13% of total labour income in Sweden and 16% in Slovenia.

## Unpaid work

By accounting only for paid work the life cycle deficit ignores a large part of production activities. In particular it gives a biased picture of the contribution of women to total production as in virtually all countries women do on average more unpaid work than men, mainly in form of unpaid household work such as childcare, cooking and cleaning (see e.g. Miranda, 2011). The output of unpaid production activities is difficult to measure and assess in physical as well as monetary terms. The physical output (e.g. number of meals prepared, kilograms of laundry washed, etc.) is usually not recorded. Furthermore, most of the goods and services which are produced through unpaid work are not traded on the market and therefore do not have a market price. For this reason unpaid work is usually valued by an “input approach”, i.e. by measuring the value of the inputs into production (see e.g. European Communities, 2013; Abraham and Mackie, 2005). Working time constitutes certainly the most important input in household production. Measures of production through unpaid work are therefore mostly based on time use surveys (for an exception see Holloway et al., 2002, who use an output approach). We also choose an input approach in our analysis and measure production through unpaid work by the amount of time which is devoted to unpaid production activities.

There are pronounced cross-country differences in the share and level of unpaid work carried out by women. These differences have been documented and analysed in a large number of comparative studies on the gendered distribution of production activities (see e.g. Gimenez-Nadal and Sevilla, 2012, for an analysis of changes over time). Some of these differences can be explained by the different institutional settings. Welfare state arrangements shape the distribution of unpaid household work by providing or denying access to resources and opportunities such as parental leave, child benefits, childcare facilities or survivor benefits. Hook (2010) for example finds that long parental leaves are positively related to gender specialization and lower contributions of men to household work. She suggests that paternity leave not only boosts the involvement in housework and childcare in the short, but also in the long run as fathers acquire skills as caretaker and the paternity leave fosters the relation between the father and children. The national context influences the level and distribution of household work also by shaping social norms and attitudes. Based on the data from the International Social Survey Program, Geist (2005) shows that in conservative welfare state regimes (Austria, Germany, Mediterranean Countries) it is more rare for couples to share housework equally than in social-democratic regimes (Scandinavian countries), which explicitly promote gender equity. Our analysis provides information on the cross-country differences in the age- and gender-specific level of unpaid work. Additionally we provide estimates of the age-specific consumption level of goods and services produced by unpaid work.

### Data: the multinational and Austrian time use survey

Our analysis of unpaid work is based on data from the Multinational Time Use Survey (MTUS)^16^ (Gershuny et al., 2012) and the Austrian time use survey from 2008.^17^ The MTUS contains data from about 60 diary based time use surveys in 20 countries. We use the surveys from the following countries: Germany (2001), Finland (1999), France (1998), Italy (2002), United Kingdom (2000),^18^ Slovenia (2000) and Spain (2002).^19^ Furthermore, we make use of the Austrian time use data from 2008, which is not yet included in the MTUS database. Unfortunately we cannot include Sweden and Hungary; the Swedish time use data does not include the necessary information on household structure and for Hungary there is no time use survey included in MTUS. Participants of time use surveys fill out diaries with predefined time slots (between 5 and 30 min) for which the respondent reports the activity he/she is carrying out during that period. The single activities were later grouped into categories of activities. As the design and the grouping of activities is different across surveys these data are harmonised within the MTUS to enable and facilitate comparisons across time and countries. Beside variables on the socio-economic background and household structure the MTUS includes the time used on the survey day(s)^20^ for 51 different categories of activities. For unpaid work we include the activity categories cook/wash up, housework (laundry, cleaning activities), other domestic work (repair, paperwork, pet care, care for adults), gardening, shopping, childcare and travel related to these activities.

### Methodology

The estimation of production, i.e. the amount of time used for unpaid work by age and gender is straight forward: we simply take the average number of minutes devoted to these production activities by gender and single years of age. The age- and gender-specific estimates for the consumption of goods and services emerging from unpaid work require assumptions about their distribution within the households. The basic assumption regarding the consumption of these goods and services (excluding childcare) is, that they are distributed within the household in equal shares, i.e. every household member consumes the same amount. Such an assumption is necessary since it is not observable how much each member of the household really consumes.^21^ To calculate the consumption of goods and services produced by household members we sum up the total time which is spent to produce these goods and services, divide it equally among all household members and calculate the average consumption level for each age group. The consumption age profiles are then adjusted so that aggregate consumption (age averages multiplied by population numbers and added up over all ages) through unpaid work equals aggregate production.

Childcare is treated differently: the bulk of childcare activities is enjoyed by the children in the first years of their life, the amount of consumption is therefore strongly dependent on the age of the child. Most time use surveys include only household members above the age of ten (France 15+, Italy 3+ and UK 8+). Furthermore, while MTUS contains a variable with the number of children, it does not contain information on the age of household members that are not included in the survey. It is therefore not possible to obtain age-specific estimates for the consumption of children. To be comparable across countries we report estimates of the production and consumption of goods and services which are produced by unpaid work only for the age groups 15+. It is assumed that childcare services are completely consumed by persons below the age of 15 years.

### Results

**Fig. 2:**
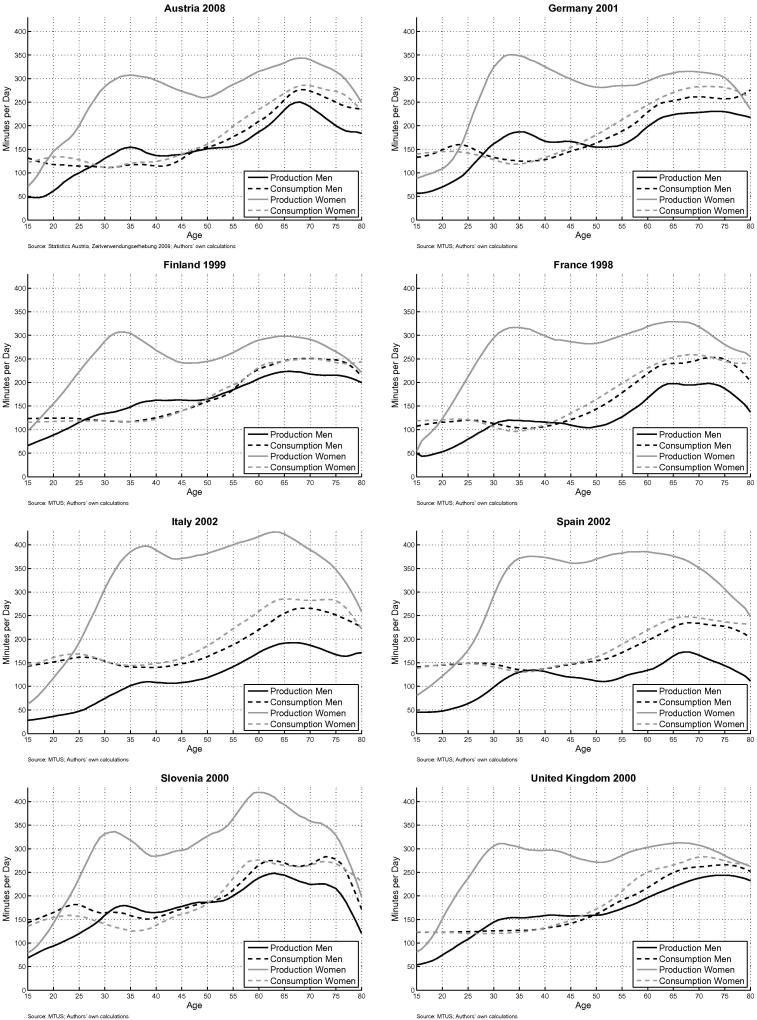
Unpaid work: production and consumption in minutes.

The averages of time devoted to unpaid work by age and sex are plotted in Fig. 2. For women the average time devoted to unpaid production activities peaks in the age group from 30 to 35 years (childcare) and in the age group from 60 to 70 years. The amount of time which is used by women for unpaid work is quite similar in Austria, Germany, Finland, France and the UK, where adult women devote on average about 5 h (300 min) daily to non-market production activities. In Spain women spend around 1 h more in non-market production activities (around 360 min) and in Italy almost two hours more than in the other countries (around 400 min). Slovenia is exceptional: like in most of the other countries there is a smaller peak in childbearing age at which women use about 5 1/2 h a day for unpaid work. However, elderly women between 55 and 70 use between 6 and 7 h for unpaid work in Slovenia, about the same as in Italy and much more than in the other European countries. For men the picture is different: from the age of 30 to about 50 they devote on average between 2 h and 2 1/2 h to unpaid work. Men do most of household work in retirement, when they devote between 3 and 4 h to unpaid work. Their contribution is over the whole age-range comparatively high in Slovenia and rather low in Italy, Spain and France.

The consumption of goods and services which are produced by household members through unpaid work is similar across gender and rather constant until the age of 50. There is a slight reduction at the age of 35, when due to the presence of children the household size is larger and household production has to be distributed over a larger number of persons. The consumption peaks in old age together with the unpaid production activities. As we assume that transfer flows in form of goods and services from unpaid work occur only within the households, intergenerational flows are only possible if several generations live together. However, in all of the countries the majority of elderly persons do not live with their children or grandchildren. The share of persons aged between the age of 60 and 70 who still live together with their children is below 10% in Finland, France and Germany, 14% in the UK, 16% in Austria, 28% in Slovenia, 35% in Italy and 40% in Spain.^22^ While in most of the countries the age profiles of male and female production add up to male and female consumption (the consumption age profiles lie between male and female production), this is obviously not the case for Slovenia. The pattern for Slovenia, where production around the age of 60 is much higher than consumption, indicates a transfer of household production goods and services from people around the age of 60 to younger generations. This is in line with information on childcare arrangements included in EU-SILC 2010, showing that in Slovenia grandparents are more involved in childcare activities than in other countries.

The difference between the consumption and production age profile represents the LCD for unpaid work. While this difference is low but positive for men between the age 30 and 50 in Austria, Germany, Finland, Slovenia and the UK, household production of men hardly exceeds their consumption in France, Spain and Italy, reflecting their low contribution to unpaid household work. In Italy the LCD for men stays positive over the whole age range. Women in turn produce more non-market goods and services than they consume with the exception of the teen ages in all countries. Their additional production is used for transfers to their children and partners. Compensating for the low contribution of men in Italy and Spain, the difference between production and consumption of women (the LCS) is much larger in these two countries than in the other European countries.

## The life cycle deficit for paid- and unpaid work

In the next step we combine production through paid and unpaid work into one single measure. The common approach is to value the time used for unpaid work by using wage rates which would be obtained on the market for similar activities (e.g. European Communities, 2003). As in MTUS the activity categories are quite general and include many different tasks, we use the same wage for all of the household production activities. The wage we apply to value unpaid work corresponds to the average hourly net income of a worker in the age group 30–49 years within a country.^23^ This approach has the advantage that we can use the same data source as for the estimates of paid work. It ensures, first, the comparability of unpaid work and paid work within a country, and second, the cross-country comparability as this is given by the EU-SILC dataset.

**Fig. 3:**
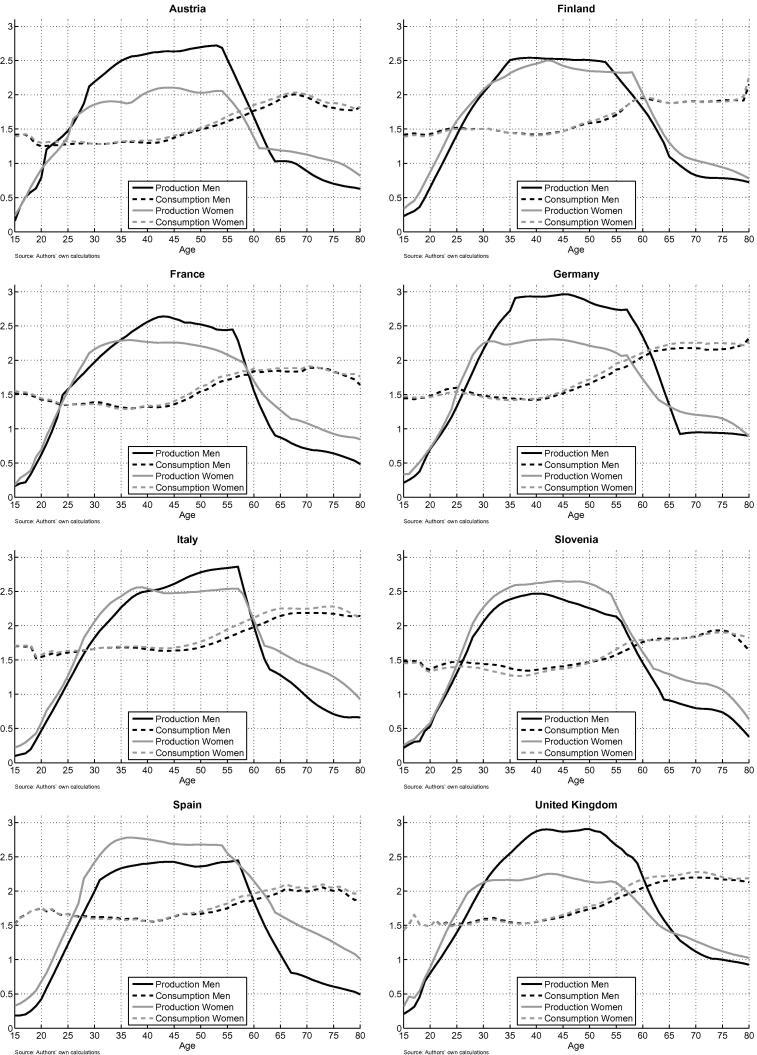
Paid and unpaid work: production and consumption by age and gender relative to the sample-average of labour income from paid work.

The measures for total production and total consumption at each age are plotted in Fig. 3. As expected, the gender differences are lower as compared to the results for paid work in Section “The life cycle deficit by gender”. According to the new measure, which includes paid and unpaid work, women in Spain and Slovenia contribute more to production than men. In Spain women devote considerably more time to production activities than men, mainly to unpaid household work. Although unpaid work is valued less (net-wage) than paid work (gross wage), Spanish women compensate for this with their higher involvement in production activities. This is easier in Spain than in the other countries, as in Spain wages are less heavily taxed and thus the valuation of unpaid work as compared to paid work is higher. Also in Slovenia women devote on average more time to production activities than men. But, contrary to Spain, Slovenian women devote almost the same amount of time to paid work as men and have almost the same average labour income as men. Nevertheless, they use more time than men for unpaid household work. As a result their total production is higher than the total production of Slovenian men. For the other countries a gender gap remains. However, this does not imply that women engage less in production activities. Indeed, in most countries women are involved in production activities to the same extent as men. The size of the gap rather depends on the female share of household work and its valuation.

Table 3 shows the combined aggregate LCS and the LCD in old age for paid and unpaid work. To allow a comparison with the results for paid work (Table 2) we measure the aggregate LCS/LCD in percent of income from paid work. The inclusion of unpaid work increases the LCS of women in all countries: it ranges from 13% of labour income in the UK to 30% in Slovenia. The LCS of men on the contrary remains nearly the same: in most of the countries men in working age produce a small LCS in terms of unpaid work, their overall LCS is therefore constant or slightly higher than in Table 2. An exception are France, Italy and Spain: the male population in working age generates a deficit in terms of unpaid work. This is reflected in the lower LCS in Table 3 as compared to Table 2 where only paid work is considered. The total LCS of men is lowest in Spain with 19% of labour income and highest in Austria and Germany with 31%. Although the gender differences within countries decrease once we include unpaid work, in most countries the LCS of women is lower than the LCS for men. The high involvement of women in unpaid work does not compensate for their low involvement in paid work, except in Spain and Slovenia. There remain large differences between countries: the total LCS (men and women) for paid and unpaid work ranges from 38% in Italy to 53% in Slovenia.

The aggregate LCD of the elderly men and women is rather constant, as most of the resources generated by the elderly through unpaid work are consumed by the elderly themselves. Although the inclusion of unpaid work has little effect on the overall LCD/LCS, there are effects on the LCD/LCD for men and women separately: the LCD of men in old age has been increasing in all of the countries, but this increase has been compensated by a decrease in the LCD of women. Women provide unpaid services not only to children but also to the elderly male household members. The age borders that separate the LCS from the LCD do not change much for the total population (men and women) if unpaid work is included.

## Conclusions

The public welfare system in the countries we considered in this paper consists to a large degree of transfers from the active population to the inactive elderly persons. Faced with population ageing and rather inflexible ages at labour market entry and exit, the funding of this system is under pressure in virtually all European countries. Our analysis highlights the fact, that the consequences of population ageing for the overall economic development and in particular for public finances do not only depend on the extent of demographic change, but are also determined by the design of the economic life cycle, i.e. by the relation between the age of individuals and the type and intensity of their economic activities. We compare selected European countries using the aggregate life cycle deficit (LCD). The LCD is a dependency ratio which takes into account not only the population structure, but also the age-specific levels of production and consumption. In order to identify the effect of the design of the economic life cycle on the dependency of children and the elderly we use country-specific age profiles of production and consumption. Due to data limitations we had to assume the same age specific consumption by gender and to assume that goods and services produced by household work are equally shared among members. Both assumptions constitute a first approximation only and further work needs to relax these restrictions. To control for the different age structures across Europe, we assume a standard population for all of the countries included in our analysis.

Our comparative analysis reveals large cross-country differences in the aggregate LCD (respectively the aggregate LCS) as a result of the differences in the design of the economic life cycle. High values of the aggregate LCD in young and old age in Italy are a consequence of the high consumption relative to labour income. Low values of the aggregate LCD in young age for Austria and Germany are driven by an early entry into the labour market and the low average consumption expenditure of children. The low value of the LCD in old age for Sweden in turn can be explained by the late exit from the labour market. Hence, the entry and exit ages to employment play an important role in determining the aggregate LCD. While in France average consumption exceeds average labour income already at age of 23, the corresponding value for Italy, the country with the highest LCD in young age, is 27. In Sweden, the country with the lowest LCD in old age, average labour income exceeds consumption until the age of 63; the corresponding age is between 57 and 59 in the other countries. Moreover it is interesting to also consider the cross-country gender differences in the life cycle surplus, a measure for the ability of the working age population to finance the LCD of the children and the elderly. These differences can be largely explained by the different shares of total labour income which are generated by women. In Slovenia and Sweden the contribution of women to total labour income is among the highest within Europe, resulting in a high LCS as compared to the other analysed countries. In Italy and the UK the LCS is low, as a consequence of the low participation of working age women in paid work.

The gender-specific analysis of the LCD/LCS is misleading if we ignore unpaid work. In all of the countries women contribute significantly more time to unpaid work than men do. The difference between production and consumption of goods and services produced by unpaid work indicates that women carry out a large part of unpaid work for men living in the same household and that the working age population carries out a large part of unpaid work for other age groups, mainly their children. Elderly persons also devote a lot of time to unpaid production activities. However, our measure of consumption indicates that these goods and services are consumed by older age groups themselves. The combination of paid and unpaid work illustrates the total dependency of elderly persons and the total contribution of the working age population to production. Even after taking into account unpaid work together with paid work there remains a gender gap in the LCS in most of the analyzed countries: men in working age usually produce more than working age women, mainly because unpaid work is valued less than paid work. However, in Slovenia and Spain women contribute considerably more time to production than men and are therefore able to compensate for the lower valuation of unpaid work.

The on-going changes in the age structure of the population require changes in the design of the average economic life cycle to maintain the fiscal sustainability of the current public transfer systems in many European countries. Our results from the cross-country comparisons suggests possible strategies how age-specific economic behaviour can be adjusted to an ageing population. The example of Sweden shows that a higher involvement of older age groups in the production process is an effective way to reduce total economic dependency of elderly persons: although Sweden has a comparably old population, the aggregate dependency of elderly persons is comparably low. Another way to adjust the funding of public transfers is through a higher involvement of working age women in paid work. The majority of women in Sweden and Slovenia is employed full time, the difference between the average labour income of men and women is low. The higher contribution of women results in a higher life cycle surplus of the working age population and resources which can be transferred to other age groups or used to accumulate assets. However, the effect of such policies on the private part of the transfer system has to be taken into account. Our results show that private transfers to children as well as the public transfers to the elderly are provided by the same age-groups. This leads to potential conflicts and trade-offs between the different types of transfers. An increase of labour force participation of working age women for example should also consider that these may have an effect on time used for unpaid work. Or most obviously, an increase the contribution of the working age-population to the public transfers system may decrease its ability to provide resources to the own children. Reforms of the transfer system need to take into account not only public transfers but also private transfers, in particular those in form of services which are produced for other household members through unpaid work.

## Figures and Tables

**Table A.1:** Generation and use of income.

	Austria	Finland	France	Germany	Italy
**Net National Income p.c. in Euro**	**22,654**	**20,509**	**19,827**	**22,294**	**17,259**
Labour Income in %	76.6	75.7	76.8	73.7	73.7
Asset Income in %	23.4	24.3	23.2	26.3	26.3
+ Transfers from ROW p.c. in Euro	−216.2	−259.5	−381.7	−418.8	−257.0
**Disposable Income**	**22,438**	**20,249**	**19,445**	**21,875**	**17,002**
Consumption in % of DI	87.5	94.6	95.3	88.8	100.5
Saving in % of DI	12.5	5.4	4.7	11.2	−0.5
Public Saving in % of DI	−2.3	−3.5	−8.2	−3.2	−5.1
Private Saving in & of DI	14.8	9.3	12.9	14.4	4.6
Consumption as Share of Labour Income	1.13	1.23	1.22	1.18	1.34

	Slovenia	Spain	Sweden	UK	Hungary
**Net National Income p.c. in Euro**	**14,594**	**17,905**	**22,264**	**21,289**	**9,905**
Labour Income in %	85.7	79.7	75.5	75.0	81
Asset Income in %	14.3	20.3	24.5	25.0	19
+ Transfers from ROW p.c. in Euro	69.6	−136.7	−423.5	−382.0	52
**Disposable Income**	**14,663**	**17,768**	**21,841**	**20,907**	**9,957**
Consumption in % of DI	93.9	96.8	83.2	98.2	95
Saving in % of DI	6.1	3.2	16.8	1.8	5
Public Saving in % of DI	−3.8	−8.8	1.8	−9.9	−9
Private Saving in & of DI	9.9	12.1	14.9	11.7	15
Consumption as Share of Labour Income	1.10	1.21	1.08	1.29	1.17

*Note:* Euro values are adjusted for differences in purchasing power.

**Table 1:** The life cycle deficit in countries.

	Aggregate Life cycle deficit/surplus in % of labour income			Age borders LCD		Standard dependency ratio	
Country	Young	Working age	Old	pos. until	pos. from	Young	Old

Austria	20	32	25	24	59	34	29
Finland	26	28	25	26	60	38	28
France	29	31	24	23	59	42	28
Germany	18	31	30	26	60	31	34
Hungary	22	32	27	24	58	33	27
Italy	26	24	32	27	60	31	33
Slovenia	24	39	24	25	58	30	26
Spain	25	27	23	26	60	31	27
Sweden	25	39	23	26	64	40	31
UK	27	23	25	26	59	40	28

*Sources*: EUROSTAT (Population); EU-SILC 2011 (Labour income); www.ntaccounts.org (Consumption).

**Table 2:** The aggregate life cycle deficit and -surplus by gender.

		Aggregate Life cycle deficit/surplus in % of labour income		
Country	Sex	Young	Working age	Old
Austria	Women	11	3	17
	Men	10	30	10
	Total	20	32	27
Finland	Women	12	9	15
	Men	12	20	10
	Total	24	29	25
France	Women	12	6	15
	Men	12	27	10
	Total	24	32	24
Germany	Women	11	2	18
	Men	10	30	10
	Total	20	30	27
Hungary	Women	11	10	18
	Men	11	23	11
	Total	23	33	29
Italy	Women	16	0	19
	Men	14	25	10
	Total	30	24	29
Slovenia	Women	14	16	17
	Men	14	23	11
	Total	28	39	28
Spain	Women	14	4	17
	Men	14	23	10
	Total	28	26	26
Sweden	Women	11	13	13
	Men	11	28	8
	Total	22	40	21
UK	Women	12	0	18
	Men	11	26	9
	Total	23	23	26

To facilitate the comparison across countries a standard population is applied. Source: Authors’ own calculations based on EU-SILC (Income) and data from the NTA project (Consumption).

**Table 3:** The life cycle surplus/deficit for paid and unpaid work.

		Aggregate			
		Lifecycle Surplus/Deficit in % of Labour Income		Age Borders LCD	
Country	Sex	Working Age	Old	pos. until	pos. from
Austria	Women	15	14	24	58
	Men	31	12	21	60
	Total	45	25	23	59
Finland	Women	21	14	23	61
	Men	22	11	25	59
	Total	42	24	24	60
France	Women	19	12	23	59
	Men	25	12	23	60
	Total	44	23	23	59
Germany	Women	16	17	24	58
	Men	31	11	26	62
	Total	47	28	25	60
Italy	Women	18	13	27	60
	Men	21	13	28	61
	Total	38	26	27	60
Slovenia	Women	30	13	24	59
	Men	23	13	26	59
	Total	53	26	25	59
Spain	Women	27	10	25	62
	Men	19	13	27	60
	Total	46	24	26	61
UK	Women	13	16	23	57
	Men	27	10	25	61
	Total	39	26	24	60

*Source:* Authors’ own calculations. *Note:* Information on the LCD for children cannot be provided, as there is insufficient information on their age in the Multinational Time Use Survey.

## Footnotes

2 *Source:* EUROPOP2013 population projection; main scenario.

3 Production includes labour income as well as unpaid household labour.

4 The young age dependency ratio relates the number of people below the age of 20 to those in working age, assumed to be the age group from 20 to 64. Similarly the elderly dependency ratio records the number of the population above age 65 relative to those in working age. Also the age borders 0–14 for children, 15–64 for the working age population and 65 + for the elderly are often used.

5 http://www.ntaccounts.org/web/nta/show/NTA%20Countries.

6 For data from Austria, Finland, Germany, Hungary, Slovenia, Spain and Sweden see Lee and Mason (2011). For the Italian data see Zannella (2013). Turkey and Poland joined the NTA project in 2012 and 2013, respectively. For these two countries no NTA dataset is available yet.

7 Transfer inflows and outflows are recorded from the individuals point of view: inflows constitute the benefits, outflows the contributions to the transfer systems. Public transfer inflows consist for example of benefits such as pensions, health services or child benefits while the public transfer outflows consist mainly of taxes and social contributions.

8 From Section “The life cycle deficit by gender” onwards we will control for the demographic structure by applying a standard population and focus on the design of the economic life course.

9 Mixed income is the surplus (or deficit) accruing from production by unincorporated enterprises; it implicitly consists of the remuneration for work done by the owner and the return for the input of the owner’s capital. Mixed income is divided into a labour share and asset share by assuming that two thirds of mixed income is labour income and one third is capital income.

10 We use the cross-sectional EU-SILC UDB - version from August 01, 2013. We herewith acknowledge data provision by EUROSTAT and the European Commission respectively. Presented results and drawn conclusions are those of the authors and not those of EUROSTAT, the European Commission or any of the national authorities whose data have been used.

11 With the exception of the UK the income reference period in the 2011 survey was the calendar year 2010. In the UK yearly income is extrapolated from smaller and flexible reference periods referring to the current year.

12 The aggregates as well as the age profile of labour income refer to 2010. The base year for the consumption profiles are as follows: Austria 2010, Finland 2004, France 2001, Germany 2003, Hungary 2005, Italy 2008, Slovenia 2004, Spain 2000, Sweden 2003, UK 2007.

13 The data for Finland, Germany, Hungary, Spain and Sweden can be downloaded from the homepage of the NTA project: www.ntaccounts.org.

14 The standardized population age structure is calculated as the average age structure of the included countries, giving each country the same weight.

15 These estimates are based on data from EU-SILC 2011.

16 This document presents results drawn from the Multinational Time Use Study (MTUS), but the interpretation of this data and other views expressed in this text are those of the authors. This text does not necessarily represent the views of the MTUS team or any agency which has contributed data to the MTUS archive. The authors bear full responsibility for all errors and omissions in the interpretation of the MTUS data.

17 Statistics Austria, Time Use Survey 2008/09 (developed on behalf of the Federal Minister for Women and Public Services)

18 The 2005 survey from the UK does not contain all the required information on the household structure.

19 This survey does not include the Basque country. The Basque survey has been carried out separately from the rest of Spain and does not include required information on the household structure.

20 While there are diaries for two days for each observation in Slovenia, Finland, the UK and Germany, it is one day in the other countries.

21 The assumption that the goods and services produced by unpaid household work are shared among the household members is simplifying also in another dimension: unpaid production can also be carried out for members of another household. While most national time use surveys include an indicator if an activity is also carried out for another household such information is not included in the version of MTUS we are using.

22 Estimates based on EU-SILC 2011.

23 The average hourly net income is calculated from EU-SILC by dividing the average weekly gross income through the average number of working hours. The gross-net conversion was made using EUROSTAT data on net earnings and tax rates. However, the information on working hours corresponds to the survey period and not necessarily to the income reference period. We restrict the analysis to the age group 30–49 years because we assume that in this group changes in the employment status between the income reference period and the survey are low. Information on the employment status during the whole income reference period is unfortunately not available for all of the countries.
